# A distal enhancer of *Pparα* regulates thermogenesis and mitochondrial function in brown fat

**DOI:** 10.1371/journal.pgen.1011915

**Published:** 2025-10-23

**Authors:** Tingting Jiang, Duo Su, Jing Ke, Xin Dai, Maohua Wang, Yan Wang, Siyuan Zhan, Tao Zhong, Jiazhong Guo, Li Li, Honping Zhang, Linjie Wang

**Affiliations:** 1 State Key Laboratory of Swine and Poultry Breeding Industry, College of Animal Science and Technology, Sichuan Agricultural University, Chengdu, Sichuan, P. R. China; 2 Key Laboratory of Livestock and Poultry Multi-omics, Ministry of Agriculture and Rural Affairs, College of Animal Science and Technology, Sichuan Agricultural University, Chengdu, Sichuan, P. R. China; 3 Key Laboratory of Agricultural Bioinformatics, Ministry of Education, Sichuan Agricultural University, Chengdu, Sichuan, P. R. China; Michigan State University, UNITED STATES OF AMERICA

## Abstract

Peroxisome proliferator-activated receptor α (PPARα) is a crucial transcription factor in regulating brown adipose tissue (BAT) physiological function. However, the mechanisms of enhancer-promoter chromatin interactions that control transcription regulation of **Ppar*α* in BAT remain unclear. In this study, we used circularized chromosome conformation capture coupled with next-generation sequencing (4C-seq) to reveal distinct differences in **Ppar*α*-associated chromatin interactions between intrascapular BAT (iBAT) and epididymal white adipose tissue (eWAT). In addition, we identified an iBAT-specific active enhancer (Pparα-En4) that was activated by cold stimulation. Functional assays demonstrated that targeted repression of Pparα-En4 region significantly decreased **Ppar*α* expression and impaired brown adipocyte differentiation and thermogenesis. Moreover, the transcription factor CREB regulated Pparα-En4 activity and increased **Ppar*α* expression in cooperation with the acetyltransferase CBP. Repression of Pparα-En4 using a lentiviral system in iBAT resulted in reduced thermogenic capacity and mitochondrial damage during cold acclimation conditions in vivo. These findings reveal that Pparα-En4 is a critical regulatory element in thermogenesis and mitochondrial function, and provide important insights into enhancer-mediated transcriptional regulation of **Ppar*α* expression in BAT.

## Author summary

Brown adipose tissue (BAT) converts chemical energy into heat through the action of mitochondrial uncoupling protein 1. The thermogenic program in adipocytes is governed by a complex network of transcription factors and associated regulatory proteins. PPARα functions as a key transcription factor involved in the thermogenic program. Enhancers serve as pivotal *cis*-regulatory elements that modulate gene expression by establishing physical interactions with their target promoters mediated by transcription factor-protein complexes. Here, we identify a BAT-specific enhancer of **Ppar*α* (Pparα-En4) that regulates thermogenesis and mitochondrial integrity of BAT. The activity of Pparα-En4 is regulated by CREB in cooperation with the acetyltransferase CBP. These findings provide important insights into enhancer-mediated transcriptional regulation in BAT.

## Introduction

Brown adipose tissue (BAT) is a specialized fat depot in mammals that is activated upon cold exposure or β-adrenergic receptor agonists [1]. The thermogenic activity of BAT is primarily facilitated by uncoupling protein 1 (UCP1), a protein specific to BAT located on the inner mitochondrial membrane [2]. In addition, activation of BAT increases glucose and lipid expenditure and plays an essential role in maintaining systemic metabolic homeostasis [3,4]. Previous studies have indicated that stimulation of thermogenic gene expression in BAT enhances whole-body energy utilization and may contribute to the prevention or reduction of obesity-related metabolic disorders [5]. Therefore, the regulation of thermogenesis in BAT presents potential opportunities for preventing obesity-related metabolic disorders.

Peroxisome proliferator-activated receptor alpha (PPARα) is best known for its role in hepatic fatty acid metabolism, where it regulates β-oxidation, fatty acid uptake, and triglyceride metabolism by directly controlling the transcription of related genes [6]. Previous studies have shown that **Ppar*α* is highly expressed in the BAT compared to white adipose tissue (WAT), serving as a marker gene distinguishing BAT from WAT [7,8], and PPARα promotes transcription of *Ucp1* [9], suggesting that PPARα may act as an important transcription factor in BAT thermogenesis. Moreover, **Ppar*α* expression is significantly upregulated following treatment with isoproterenol (ISO) in mouse brown adipocytes [10]. Pharmacological inhibition of PPARα using GW6471 attenuates BAT thermogenesis and lowers ATP levels during acute cold exposure in vivo [11]. PPARα-BATKO mice exposed to 4°C for 24 hours do not display a pronounced defect in UCP1 expression and rates of VO_2_ and VCO_2_ [12]. However, the role of PPARα in BAT thermogenesis during long-term cold adaptation remains to be elucidated. Collectively, these findings establish PPARα as a pivotal regulator of brown adipocyte thermogenesis. Nevertheless, the transcriptional regulatory mechanisms controlling **Ppar*α* expression in BAT remain poorly understood.

Enhancers are essential *cis*-regulatory elements that control gene expression by forming physical contacts with their target promoters through transcription factor-mediated protein interactions [13–15]. Functionally active enhancers are typically associated with open chromatin marked by histone modifications such as H3K27ac and H3K4me1, as well as the transcriptional coactivator CBP [16–18]. In BAT, few studies have explored the role of enhancers in regulating adipose function. Peroxisome proliferator-activated receptor gamma coactivator 1alpha (PGC1α) has been reported to bind to the enhancer region of **Cebp*α* in response to acute cold exposure [19]. Our recent study identified a distal enhancer of *Ucp1*, that EBF2 binding regulate thermogenic capacity and mitochondrial function in BAT [20]. Given the critical role of PPARα in brown fat development and thermogenesis, it is essential to identify the functional enhancers that regulate **Ppar*α* expression and to elucidate the underlying mechanisms of its transcriptional regulation.

In this study, we employed circularized chromosome conformation capture coupled with next-generation sequencing (4C-seq) to characterize the difference in chromatin interactions of **Ppar*α* between interscapular BAT (iBAT) and epididymal WAT (eWAT). Our analysis identified three active enhancers in iBAT and further explored the function of Pparα-En4 both in vivo and in vitro. Functional validation demonstrated that Pparα-En4 modulated **Ppar*α* expression, thereby influences brown adipocyte differentiation and thermogenesis. In vivo lentiviral injection into iBAT revealed that Pparα-En4 is essential for maintaining thermogenic capacity and mitochondrial integrity during cold adaptation. These findings elucidate important mechanisms underlying the transcriptional regulation of **Ppar*α* and provide important insights into the development of targeted therapies for obesity and related metabolic disorders.

## Results

### Characterization of *Pparα* chromatin interactomes between iBAT and eWAT

**Fig 1:**
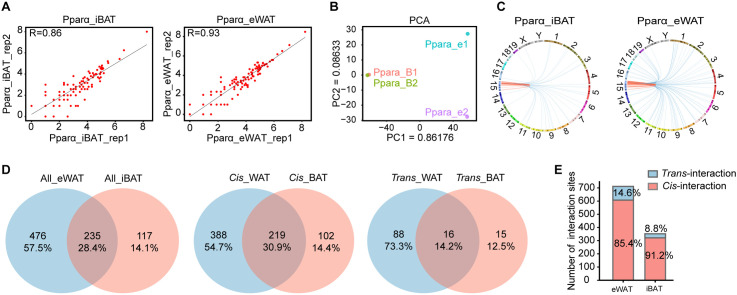
Characterization of *Pparα* chromatin interactomes between iBAT and eWAT. **(A)** Scatter plot showing **Ppar*α* chromatin interaction sites distribution in interscapular BAT (iBAT) and epididymis WAT (eWAT). **(B)** PCA showing the **Ppar*α* chromatin interactions in iBAT and eWAT. **(C)** Circos plot illustrating genome-wide interactions of **Ppar*α*. **(D)** Venn diagram displaying the number of shared and tissue-specific chromatin sites of **Ppar*α*. **(E)** Histogram showing the proportion of *cis*-interaction and *trans*-interaction sites in iBAT and eWAT.

To investigate the differences in **Ppar*α* chromatin interaction patterns between iBAT and eWAT, we performed 4C-seq to analyze the regions that interact with the **Ppar*α* promoter region (−2000 bp to +500 bp) (S1A Fig). Analysis of the *cis*/*trans* interaction ratio across four 4C datasets showed that 60%-77% of mapped reads were located on the same chromosome (S1B Fig and S1 Table). These results met the quality control criterion of ‘*cis*/overall ratio of > 40%’ [21], confirming the reliability and high quality of the 4C experiments. The genome-wide interaction sites of **Ppar*α* were determined using a continuous non-overlapping 2 kb window method (S2 Table). A high degree of inter-replicate concordance was observed (Pearson’s r = 0.86 for iBAT, 0.93 for eWAT; Fig 1A). Principal component analysis revealed that chromatin interactions of **Ppar*α* in iBAT and eWAT showed distinct group (Fig 1B), suggesting distinct chromatin interaction profiles of **Ppar*α* between iBAT and eWAT.

We identified 352 iBAT-specific and 711 eWAT-specific **Ppar*α* interaction sites (Fig 1C and S3 Table), along with 235 shared regions (16 *trans*-interaction, 219 *cis*-interaction; Fig 1D) by analyzing the interaction sites shared between two replicates. Notably, the number of interaction sites in eWAT was approximately twice that observed in iBAT. Moreover, over 90% of iBAT interaction sites and 85.4% of eWAT interaction sites were located on the same chromosome as the viewpoint (**Ppar*α* promoter region, −2000 bp to +500 bp, Fig 1E), suggesting that the activation of *cis*-regulatory elements may contribute to the tissue-specific expression of **Ppar*α* in iBAT.

### Identification of *Pparα* active enhancers under cold stimulation

**Fig 2:**
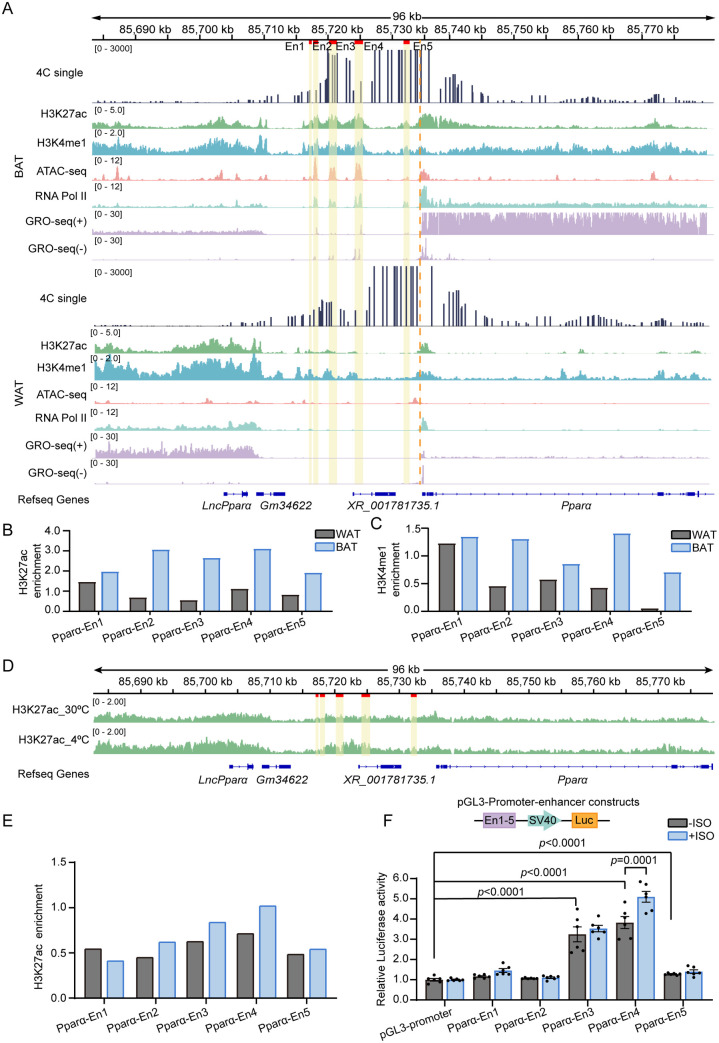
Identification of *Pparα*active enhancers. **(A)** IGV visualization of manually selected potential **Ppar*α* enhancers. The yellow line indicating the **Ppar*α* enhancer viewpoint. **(B, C)** Histone modification signal intensities for H3K27ac (B) and H3K4me1 (C) at potential **Ppar*α* enhancers regions. Signal values were obtained from IGV. **(D)** IGV visualization of H3K27ac ChIP-seq at **Ppar*α* potential enhancers in BAT under 30°C and 4°C for 2 days. The yellow line indicating the Pparα enhancer viewpoint. **(E)** Histone modification signal intensities for H3K27ac at potential Pparα enhancers regions. Signal values were obtained from IGV. **(F)** Top: the schematic diagram of the luciferase reporter construct used for enhancer detection; Dual-luciferase reporter assay of **Ppar*α* putative enhancers activity with vehicle or 10 μM ISO treatment for 4 h. Data are shown as mean ± SEM (n = 6). All statistical analyses were performed two-way ANOVA followed by Tukey’s test.

To systematically identify potential BAT-specific active enhancers of **Ppar*α* in iBAT, we integrated 4C-seq and several publicly accessible enhancer-associated datasets, including H3K27ac, H3K4me1, ATAC-seq, RNA Pol Ⅱ, and GRO-seq profiles. GRO-seq, a popular method for detecting nascent RNA, was employed to identify active enhancers [22,23]. Comparative analysis revealed five tissue-specific putative active enhancers exclusively in iBAT (Fig 2A). These enhancer regions exhibited significant co-enrichment across histone modification markers (H3K27ac/H3K4me1), ATAC-seq, and GRO-seq. H3K27ac and H3K4me1 histone modifications of five enhancers were higher in iBAT than that in eWAT (Fig 2B and 2C). Next, we cloned these enhancers and performed luciferase reporter assays in differentiated brown adipocytes to evaluate the enhancer activity of **Ppar*α* (Fig 2F). Among them, Pparα-En3 (~3.2-fold), Pparα-En4 (~3.8-fold), and Pparα-En5 (~1.8-fold) exhibited significant transcriptional activation.

To evaluate whether the putative enhancers are activated under cold exposure, we analyzed published H3K27ac ChIP-seq data from iBAT of mice housed at 30°C or 4°C for 2 days. Cold exposure induced higher H3K27ac enrichment at Pparα-En2, Pparα-En3, and Pparα-En4 (Fig 2D and 2E), indicating cold-induced activation of these enhancers in vivo. To further investigate whether enhancers respond to β-adrenergic stimulation, we examined their activity upon ISO treatment. Among the three enhancers, only activity of Pparα-En4 (~1.33-fold) exhibited a significant increase, while the other two enhancers showed no notable activation (Fig 2F). Therefore, we subsequently performed functional validation of Pparα-En4 in BAT following cold treatment.

### Identification of functional enhancers regulating *Pparα* expression and affecting brown adipocyte thermogenesis under cold stimulation

To investigate the effect of the Pparα-En4 on brown adipocytes, we first examined **Ppar*α* expression during brown adipocyte differentiation. The results showed that **Ppar*α* expression progressively increased throughout brown adipocyte differentiation, reaching its highest level on day 8 (S2A Fig). Furthermore, **Ppar*α* expression (2.8-fold) was significantly upregulated following ISO treatment (S2B Fig). Next, we employed the dCas9-KRAB epigenetic system to specifically silence Pparα-En4. To verify the expression of dCas9-KRAB system, we performed transient transfection of the dCas9-KRAB vector on day 4 of differentiation. The results of Cas9 mRNA and protein level confirmed successful expression of the dCas9-KRAB system (S2C and S2D Fig).

**Fig 3:**
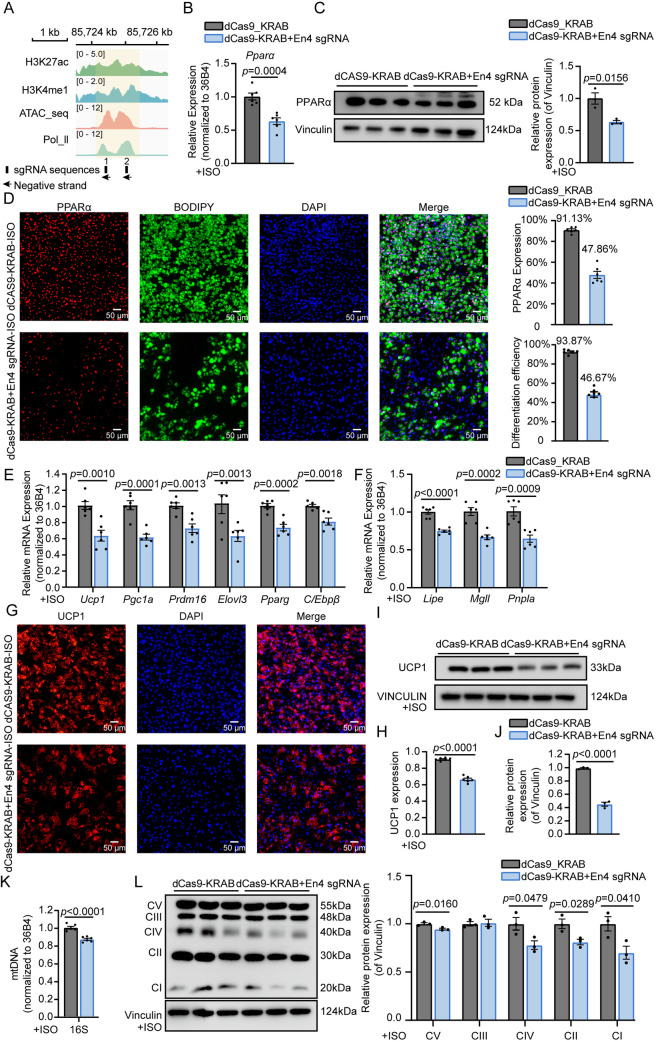
Identification of functional enhancers regulating *Pparα* expression and affecting brown adipocyte thermogenesis under cold stimulation. **(A)** Schematic representation of the dCas9-KRAB system targeting the Pparα-En4 regions. Enhance regions are highlighted in yellow, sgRNA target sites are marked in black. (**B, C**) qRT-PCR (n = 6) and western blot (n = 3) analysis of PPARα expression in brown adipocytes transduced with dCas9-KRAB+En4 sgRNA compared with control, band intensity was analyzed using ImageJ software. **(D)** Immunofluorescence staining of PPARα (red), lipid droplets (BODIPY, green), nuclei (DAPI, blue) on the day 8 of adipocyte differentiation. The percentage of PPARα-positive cells and differentiation efficiency were quantified using Image J. Differentiation efficiency was calculated by randomly selecting six microscopic fields per well, counting the total number of cells and the number of differentiated adipocytes. Scale bar: 50 μm. (**E, F**) qRT-PCR analysis of genes related to brown adipocyte function (E: *Ucp1*, *Pgc1a*, *Prdm16*, *Elovl3*, *Parag* and **C/Ebp*β*) and lipolysis-related genes (F: *Lipe*, *Mgll* and *Pnpla*). **(G)** Immunofluorescence staining of UCP1(red) and nuclei (DAPI, blue) in brown adipocytes. Scale bar: 50 μm. **(H)** The expression of UCP1 was quantified using Image J. **(I, J)** Western blot analysis of UCP1 protein level (n = 3), band intensity was analyzed using ImageJ software. (K) qRT-PCR analysis of mitochondrial DNA (16S) level in brown adipocytes. **(L)** Western blot analysis of OXPHOS complex subunits (n = 3), band intensity was analyzed using ImageJ software. Data are shown as mean ± SEM.

Two single guide RNAs (sgRNAs) were designed to target the Pparα-En4 region (Fig 3A). The expression of **Ppar*α* was significantly repressed compared to dCas9-KRAB control with ISO treatment (Fig 3B). Consistently, the protein of PPARα was significantly reduced (Fig 3C). Immunofluorescence analysis showed that repression of Pparα-En4 significantly reduced PPARα expression with ISO treatment (Fig 3D). These findings suggested that the repression of **Ppar*α* enhancers impaired PPARα expression. BODIPY staining further indicated that repression of Pparα-En4 led to a decrease in adipocyte differentiation (Fig 3D). Meanwhile, the expression levels of thermogenesis related genes, including **Pgc1*α* (~0.6-fold), *Prdm16* (~0.7-fold), *Elovl3* (~0.6-fold), **Ppar*γ* (~0.7-fold), and **C/Ebp*β* (~0.8-fold), were significantly decreased following dCas9-KRAB-En4 transfection (Fig 3E), whereas *Abhd5*, *Cidea*, and *Dio2* remained unchanged (S2E Fig). Furthermore, the expression levels of lipolysis-related genes, including *Lipe* (~0.7-fold), *Mgll* (~0.6-fold) and *Pnpla* (~0.6-fold) were also significantly downregulated (Fig 3F).

Subsequently, repression of Pparα-En4 reduced *Ucp1* mRNA expression and protein levels in brown adipocytes (Fig 3I and 3J). Compared to dCas9-KRAB-ISO control, immunofluorescence staining showed a marked decrease in UCP1 fluorescence intensity in dCas9-KRAB+En4 sgRNAs cells (Fig 3G and 3H). Following Pparα-En4 repression, mtDNA levels were significantly (~0.8-fold) reduced compared to controls (Fig 3K). To further investigate mitochondrial function, we examined the expression levels of oxidative phosphorylation (OXPHOS) complex subunits. Under ISO stimulation, repression of Pparα-En4 resulted in a marked decrease in expression of several OXPHOS complex subunits, including complex I (NDUFB8), complex II (SDHB, ~ 0.8-fold), complex IV (MT-CO1, ~ 0.7-fold) and complex V (ATP5A, ~ 0.7-fold) (Fig 3L). These findings demonstrate that Pparα-En4, as a functional enhancer, regulates PPARα expression and thereby affecting thermogenesis and mitochondrial function in response to cold stimulation.

### CREB cooperates with CBP to regulate the activity of Pparα-En4 to regulate UCP1 expression

**Fig 4:**
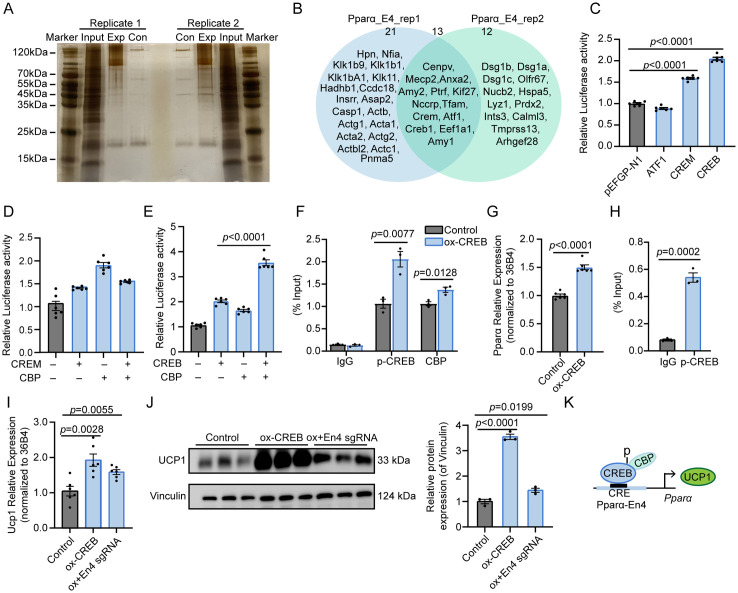
CREB cooperates with CBP to regulate the activity of Pparα-En4. **(A)** Silver staining of nucleoproteins isolated by DNA pull-down assay targeting the Pparα-En4 region. **(B)** Venn diagram illustrating the overlap of transcription factors identified in two biological replicates. **(C)** Luciferase reporter assay measuring Pparα-En4 transcriptional activity in HEK293T cells co-transfected with pEGFP-N1 (control), ATF1, CREM, or CREB expression constructs. **(D)** Quantification of relative luciferase activity in HEK293T cells co-transfected with Pparα-En4 reporter and overexpression vectors encoding CREM, CBP, or combination of CREM and CBP. **(E)** Luciferase assay evaluating Pparα-En4 activity upon co-transfection with CREB, CBP, or their combination in HEK293T cells. **(F)** ChIP-qPCR analyses revealing that both phosphorylation CREB (p-CREB) and CBP were enriched at the Pparα-En4 region transfection with control or CREB-overexpressing (ox-CREB) in brown adipocytes (n = 3). (**G**) qRT-PCR showing **Ppar*α* expression in brown adipocytes following ox-CREB compared to control (n = 6). **(H)** ChIP-qPCR analyses revealing that phosphorylation CREB (p-CREB) was enriched at the Pparα-En4 region in iBAT under room temperature (n = 3). (**I**) qRT-PCR showing *Ucp1* expression in brown adipocytes following ox-CREB or ox-CREB+En4 sgRNA (dCas9-KRAB+En4 sgRNA) compared to control (n = 6). **(J)** Western blot (n = 3) analysis of UCP1 expression in brown adipocytes transduced with ox-CREB or ox-CREB+En4 sgRNA compared with control, band intensity was analyzed using ImageJ software. **(K)** Schematic summary: CBP is recruited to combine with p-CREB, enhancing Pparα-En4 transcription activity. CREB and PPARα enhancing Ucp1 expression. Data are shown as mean ± SEM.

To investigate the transcription factors (TFs) involved in the regulation of Pparα-En4 activity, we conducted a DNA pulldown assay using nuclear extracts of iBAT. The proteins interacting with the Pparα-En4 region were subsequently resolved by SDS-PAGE gel electrophoresis (Fig 4A and S7 Table). Mass spectrometry analysis of two independent biological replicates identified a total of 13 TFs that specifically interacted with the Pparα-En4 region (Fig 4B). Interestingly, we identified all three members of the cAMP-responsive element-binding (CREB) family (CREB, ATF1, CREM) (Fig 4B). Luciferase report assays revealed that overexpression of ATF1 had no effect on Pparα-En4 activity, while Pparα-En4 activity was significantly increased upon CREM (~1.5-fold) and CREB (~2.0-fold) overexpression, respectively (Fig 4C). The transfection efficiency was evaluated by GFP fluorescence imaging, and the expression levels of *Crebbp*, *Atf1*, *Crem*, and *Creb* are shown in S3A and S3B Fig. Protein kinase A (PKA) phosphorylates CREB family, thereby activating the transcriptional program of thermogenic gene in brown adipocytes [24]. CREB-binding protein (CBP), a histone acetyltransferase and transcription coactivator, which can enhancer transcription through interaction with phosphorylation CREB and CREM [25,26]. We hypothesized that the recruitment of CREB/CREM and CBP modulated the activity of Pparα-En4, thereby enhancing **Ppar*α* transcription. To validate this, CREB/CREM and CBP overexpression vectors were co-transfected with pGL3-promoter-En4 plasmid into H293T cells. Co-transfection with CREM and CBP overexpression vectors did not enhance Pparα-En4 activity (Fig 4D). Notably, co-transfection with CREB and CBP overexpression vectors markedly enhanced Pparα-En4 activity (~3.5-fold), indicating an additive role of CREB and CBP in Pparα-En4 activation (Fig 4E). ChIP-qPCR analysis further confirmed that CREB overexpression significantly enhanced the binding of CREB (~2.0-fold) and CBP (~1.3-fold) to Pparα-En4 region (Fig 4F). Additionally, the expression of **Ppar*α* was markedly increased following CREB overexpression (Fig 4G).

To further examine CREB regulation of Pparα-En4 in vivo, we performed p-CREB ChIP-qPCR in BAT and observed significant enrichment of CREB at Pparα-En4, confirming it as a direct CREB-binding site (Fig 4H). We next examined whether CREB regulates UCP1 expression by activating **Ppar*α*. CREB overexpression alone robustly increased UCP1 mRNA (~1.9-fold) and protein (~3.5-fold) expression, whereas co-transfection with dCas9-KRAB+En4 sgRNAs markedly attenuated this induction (Fig 4I and 4J). These findings indicate that CREB-mediated *Ucp1* activation is at least partly dependent on **Ppar*α* transcriptional regulation. Together, our results establish that CREB, in cooperation with CBP, is recruited to the Pparα-En4 enhancer to drive **Ppar*α* expression, thereby indirectly contributing to *Ucp1* induction (Fig 4K).

### Repression of Pparα-En4 affects thermogenesis in iBAT

**Fig 5:**
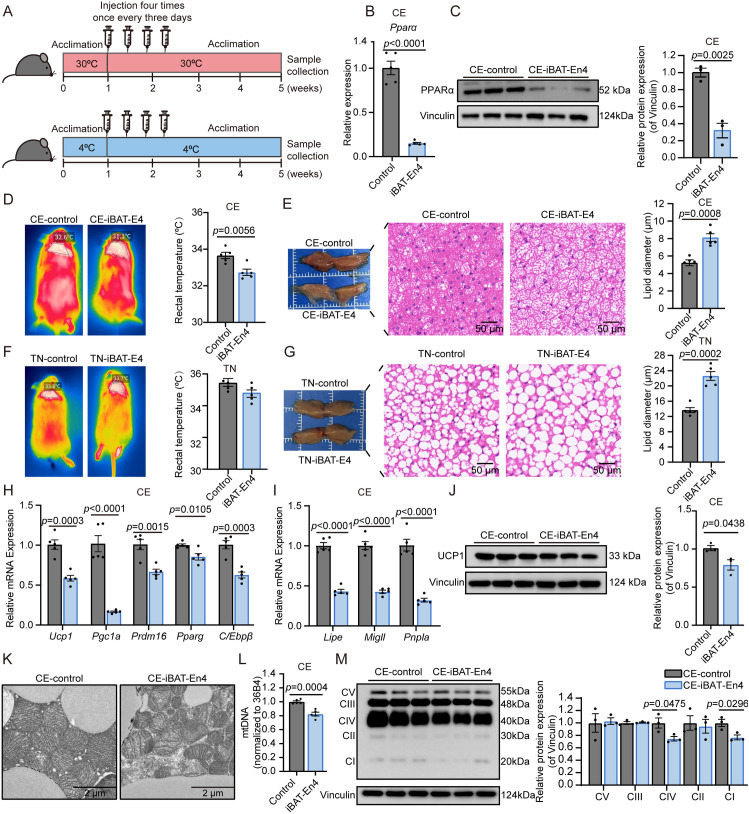
Repression of Pparα-En4 affects thermogenesis in iBAT. **(A)** Schematic overview of the lentiviral injection experimental timeline and temperature treatment into iBAT (syringe image from Bioicons, https://bioicons.com). (**B, C**) qRT-PCR (n = 5) and western blot (n = 3) analysis of PPARα expression, band intensity was analyzed using ImageJ. **(D, F)** Representative infrared images and rectal temperature at cold exposure (CE, 4°C) and thermoneutrality (TN, 30°C). **(E, G)** Representative images (left) and H&E staining (right) of iBAT from two groups at CE or TN. Scale bar: 50 μm. (**H,** **I)** Genes quantification related to brown adipocyte function (H: *Ucp1*, *Pgc1a*, *Prdm16*, *Parag* and **C/Ebp*β*) and lipolysis-related genes (I: *Lipe, Mgll and Pnpla*) by qRT-PCR (n = 5). **(J)** Western blot analysis of UCP1 (n = 3), band intensity was analyzed using ImageJ. **(K)** TEM images of iBAT mitochondrial. Scale bar = 2 μm. (L) mtDNA quantification by qRT-PCR at CE (n = 5). **(M)** Western blot analysis of OXPHOS complex subunits in iBAT at CE (n = 3). Band intensity was analyzed using ImageJ. Data are shown as mean ± SEM.

To determine whether Pparα-En4 affects the function of iBAT, dCas9-KRAB lentiviral system was administered directly into iBAT. Mice were maintained at either cold exposure (CE, 4°C) or thermoneutrality (TN, 30°C), receiving lentiviral injection four times, once every three days (Fig 5A). Immunofluorescence analysis validated the effective injection of lentivirus in iBAT (S4A and S4B Fig). We subsequently evaluated **Ppar*α* expression in iBAT from both control and iBAT-En4 groups. The results demonstrated a significant decrease in **Ppar*α* mRNA expression at both CE (~0.15-fold) and TN (~0.5-fold) (Figs 5B and S4C). Consistently, PPARα protein expression was significantly reduced at both CE (~0.3-fold) and TN (~0.5-fold) (Figs 5C and S4D). These results indicate that repression of Pparα-En4 effectively reduces PPARα expression in iBAT.

To investigate the physiological role of Pparα-En4 in vivo, we employed a lentiviral-based enhancer repression system into mouse iBAT. The results showed that the body weight of the control group and iBAT-En4 mice did not change significantly at either CE or TN (S4E Fig). However, thermal imaging showed that surface temperature in the interscapular area and the rectal temperature were markedly lower in iBAT-En4 mice at CE, while no notable difference was detected under thermoneutral conditions (Fig 5D and 5F). H&E staining further demonstrated an enlargement of lipid droplets in iBAT from iBAT-En4 mice at both temperatures (Fig 5E and 5G). In addition, *Ucp1* mRNA expression and protein levels were markedly reduced in iBAT-En4 mice upon cold exposure (Fig 5H and 5J). Notably, UCP1 protein levels remained unchanged in iBAT-En4 mice at TN (S4F Fig). We observed the expression level of lipolysis-related genes, including *Lipe* (~0.7-fold) and *Pnpla2* (~0.6-fold) was significant downregulation, indicating impaired lipolysis capacity may underlie lipid accumulation in BAT at TN (S4G Fig). The key thermogenic genes associated with brown fat, including **Pgc1*α* (~0.3-fold), *Prdm16* (~0.7-fold), **Ppar*γ* (~0.8-fold), and **C/Ebp*β* (~0.7-fold), were significantly decreased from iBAT-En4 mice at CE (Fig 5H). Furthermore, the expression of lipolysis-related genes, including *Lipe* (~0.6-fold), *Mgll* (~0.5-fold) and *Pnpla* (~0.4-fold) were also significantly downregulated (Fig 5I). Together, these findings suggest that repression of Pparα-En4 impacts the iBAT thermogenic function under cold acclimation conditions, but not at TN.

To assess the potential impacts of Pparα-En4 repression on mitochondrial structure and function, we performed transmission electron microscopy (TEM). We found that mitochondria in iBAT-En4 mice appeared swollen with irregular cristae at CE, while no changes were observed at TN (Figs 5K and S4H). Additionally, a significant reduction in mtDNA levels, along with decreased expression of complex I (NDUFB8) and complex V (ATP5A), was observed in iBAT-En4 mice under cold exposure, but not at TN (Figs 5l, 5M, S4I and S4J). These findings indicate that Pparα-En4 repression impairs mitochondrial structure and function in iBAT during cold acclimation conditions.

## Discussion

BAT dissipates energy through an uncoupled respiration process mediated by UCP1, leading to fatty acid oxidation and increased thermogenesis. BAT protects mammals from hypothermia through non-shivering thermogenesis [27]. Moreover, enhancing energy expenditure via BAT activation has the potential to counteract human metabolism disease, such as insulin resistance, obesity, and type 2 diabetes [28–31]. The thermogenic program in adipocytes is regulated by a complex network of transcription factors and associated regulatory proteins. Among them, PPARα, a member of the steroid hormone receptor superfamily, function as an important regulator of this process [32]. Although previous studies have established the important role of **Ppar*α* in BAT thermogenesis, the identification and functional characterization of its enhancers remain largely unexplored. In this study, we employed 4C-seq to characterize the **Ppar*α*- specific chromatin interactions in iBAT and eWAT. By integrating public data, we identified five potential active enhancers of **Ppar*α* in iBAT. Functional assays further demonstrated that Pparα-En4 regulates **Ppar*α* expression, as well as mitochondrial function and thermogenic activity in brown adipocytes and iBAT.

Previous studies have demonstrated a positively correlation between changes in gene expression and promoter interaction sites [33]. 4C-seq is a one-to-all technique to explore the interactions between a special chromatin region and global chromatin profiles [34]. It has been widely used to study enhancer-promoter communication, as it provides evidence by confirming physical interactions between gene promoters and putative enhancers [35]. In this study, we employed 4C-seq to map the chromatin interaction profiles of **Ppar*α* in both iBAT and eWAT. Our results revealed that iBAT exhibited more *cis*-chromatin interactions compared to *trans*-chromatin interactions. Considering the **Ppar*α* expression in iBAT is higher compared with eWAT, these results suggest that its expression may be regulated by cis regulatory elements in iBAT. Histone modification of chromatin and chromatin accessibility are known to influence gene expression [36,37]. Chromatin regions interacting with the **Ppar*α* promoter in iBAT displayed higher chromatin activity (H3K27ac and H3K4me1), suggesting these regions may be active. By integrating 4C-seq data with other epigenetic datasets, we identified five potential active enhancers regions of **Ppar*α* that exhibit elevated levels of active histone marks in iBAT. These regions, characterized by open chromatin, may facilitate transcription factor to regulate **Ppar*α* expression. Dual-luciferase reporter assays further demonstrated that three of these enhancer regions exhibited significantly higher transcriptional activity. Upon cold exposure, PPARα is activated by lipid ligands generated through β-adrenergic-induced lipolysis [38]. To further investigate whether the activity of **Ppar*α* enhancers was induced by cold stimulation, brown adipocytes were treated with ISO (a β-adrenergic receptor agonist). We observed a significant increase in Pparα-En4 activity, while the other two enhancers showed no notable activation after ISO treatment. For Pparα-En3, although it appears high basal activity, no changes were observed following ISO treatment. These results indicate that *Ppar*α-En3 act mainly as a constitutive enhancer, maintaining basal **Ppar*α* expression during adipocyte differentiation rather than responding to β-adrenergic stimulation. This finding is consistent with recent research indicating that the relationship between enhancers and promoters are dynamically regulated depending on cell type and physiological conditions [39].

Additionally, previous studies have revealed that **Ppar*α* super enhancer-driven noncoding RNAs promote the accumulation of histone demethylase KDM4B, which reduces H3K9me3 levels at the **Ppar*α* promoter and facilitates its transcriptional activation in human cardiomyocytes [12,40]. Although the human PPARα-associated seRNA shares 45.6% nucleotide identity with the mouse homolog (**lncPpar*α*), it shows lower chromatin accessibility and chromatin accessibility compared to the Pparα-En4. These findings suggest that **Ppar*α* expression is regulated by distinct *cis*-regulatory elements across different species and tissues. As a nuclear receptor and transcription factor, PPARα can directly bind to PPREs (PPAR response elements) to regulate the genes expression (*Cpt1b*, *Acox1*, and *Acadl*), which involved in fatty acid oxidation and mitochondrial metabolism [41]. Indirectly, PPARα enhances the thermogenic program by activating PGC1α and cooperating with PRDM16. In brown adipocytes, PPARα activates **Pgc1*α* transcription, while PRDM16 interacts with PPARα at the **Pgc1*α* promoter to amplify this induction, particularly under β-adrenergic stimulation through the cAMP/PKA pathway [42]. In this study, many effects observed, such as in *Ucp1*, **Pgc1*α*, and *Prdm16*, were quantitatively modest (~0.6-0.8-fold). Previous studies have demonstrated that changes of less than 2-fold can still hold biological significance and are enriched in critical pathways [43]. Furthermore, master regulators such as PGC1α and PRDM16 act as transcriptional hubs, where even minor alterations in expression can elicit amplified downstream effects on mitochondrial biogenesis and thermogenic programs [44].

Upon cold stress, norepinephrine released through the sympathetic nervous system binds to adrenergic receptors on brown adipocytes [45]. The stimulation increases intracellular cAMP level and activates protein kinase A (PKA). Activated PKA then phosphorylates CREB, thereby activating thermogenic gene program. Phosphorylation of CREB at Ser133, a key phosphor-acceptor site, promotes *Ucp1* transcription by recruitment of co-activator CBP [24]. Moreover, CBP, a histone acetyltransferase responsible for writing the transcriptionally activating mark H3K27ac [46]. DNA pulldown results revealed that CREB was identified on Pparα-En4 region. Given this, we hypothesized that CREB might also regulate **Ppar*α* transcription. ChIP-qPCR analysis demonstrated CREB enrichment at the Pparα-En4 region in vivo and in vitro, supporting its direct regulatory role at this enhancer. Consistently, CREB overexpression increased **Ppar*α* and *Ucp1* expression in brown adipocytes, underscoring a dual role of CREB in thermogenic gene regulation. Consistent with previous reports that CREB directly regulates *Ucp1* via enhancer binding [47,48]. Together, these findings indicate that CREB, in cooperation with CBP, is recruited to *Ppar*α-En4 to promote **Ppar*α* transcription, thereby indirectly contributing to *Ucp1* regulation.

Previous studies have shown that inhibition of PPARα decreases UCP1 expression in vitro, whereas short-term cold exposure (4 h and 24 h) has no effect on the UCP1 expression and oxygen consumption rate in vivo [12,49]. In this study, we found that PPARα is required for maintaining mitochondrial function and sustained thermogenesis in BAT during long-term cold adaptation. Under acute stimulation, lipolysis is rapidly activated in brown adipocytes, releasing fatty acids that directly bind to and activate UCP1 to drive thermogenesis. In contrast, during long-term cold adaptation, PKA signaling induces transcription factors such as PGC1α, which in turn promote *Ucp1* transcription and support sustained thermogenesis [50]. Thus, while PPARα plays only a minor role during acute cold responses, it becomes critical for long-term cold adaptation in BAT. Previous studies have found that **Ppar*α* knockout mice exhibit increased lipid accumulation in BAT [51]. In our results, lipid droplet accumulation in iBAT was increased in iBAT-En4 mice compared to controls, and the expression of UCP1 and mitochondrial respiratory complexes was significantly decreased. Upon cold exposure, UCP1 is activated and dissipates the proton gradient to produce heat [52]. Consequently, the downregulation of mitochondrial complexes suggests impaired thermogenic capacity in BAT. TME analysis revealed that the iBAT exhibited swollen mitochondria with disrupted cristae at 4°C after repression of PPARα-En4. In our results, both protein levels of OXPHOS complex and mtDNA content were significantly reduced after repression of Pparα-En4 at 4°C. In particular, the expression of complex Ⅰ (NDUF88) and complex Ⅳ (MTCO1) were markedly reduced. These findings suggest that Pparα-En4 repression under cold exposure disrupts mitochondrial integrity and function in iBAT. Under thermoneutral conditions, BAT activity is substantially suppressed, with reduced UCP1 expression, enlarged lipid droplets, and uptake of TAG-rich lipoproteins as well as reduced de novo lipogenesis [53–55], reflecting low energy demand. In thermoneutral conditions, the regulatory effect of PPARα on UCP1 is limited. Nevertheless, repression of Pparα-En4 under thermoneutrality still resulted in enlarged lipid droplets and downregulation of lipolysis-related genes (*Lipe* and *Pnpla2*), indicating impaired lipolysis process independent of UCP1-mediated thermogenesis.

In summary, we identified a functional enhancer of **Ppar*α* and characterized the role of Pparα-En4 in regulating thermogenesis and mitochondrial function in brown fat in response to cold. These findings offer new insights into enhancer-promoter interactions that control the transcriptional regulation of thermogenic genes in brown fat.

## Materials and methods

### Ethics statement

All animal experiments were conducted according to the Regulations for the Administration of Affairs Concerning Experimental Animals (Ministry of Science and Technology, China, revised in March 2017) and approved by the Animal Ethical and Welfare Committee (AEWC) of Sichuan Agricultural University under permit No. DKY-B2024102002.

### 4C-seq assay

4C-seq was conducted on mice iBAT and eWAT following previously established protocols [35]. The sequencing libraries were prepared using a two-step enzyme digestion and two-step PCR. Experiments targeting the **Ppar*α* gene were performed on iBAT and eWAT from C57BL/6 male mice aged 8 weeks (n = 2) at room temperature (RT). Adipose tissue (1 g) was ground into powder in a mortar and fixed with 2% formaldehyde for 30 min. Then, the crosslinked cells were lysed in 1 mL cold lysis buffer on ice for 10 min. The crosslinked chromatin was digested with *Dpn*Ⅱ (NEB) and *Csp*6Ⅰ (Thermo Fisher Scientific) for first and second digestion. T4 DNA ligase (NEB) was employed to perform ligation overnight at 16°C. DNA was purified to obtain the 4C libraries. A total of 3.2 μg DNA was used as the template for 4C PCR, divided into 16 reactions. PCR products between 200–800 bp were separated via 2% agarose gel electrophoresis, and the appropriate bands were excised. The 4C-seq libraries were sequenced using the Illumina NovaSeq 6000 platform (Illumina). The primer sequences for 4C-seq are provided in S4 Table.

### 4C-seq data analysis

Sequencing reads were demultiplexed, quality-trimmed, and aligned using the pipe4C pipeline [56]. The r3Cseq packages [57] were utilized for downstream analysis of the 4C-seq data. We mapped the trimmed sequences to the mm10 mouse genome using Bowtie2 (v2.2.5). Reads aligning to genomic regions flanking the *Dpn*II and *Csp*6Ⅰ restriction sites were defined as 4C fragment ends. Interaction counting and normalization were executed using r3Cseq to identify interaction regions in a 2-kb non-overlapping sliding window. All software commands and workflow configuration files used for 4C-seq data analysis have been deposited in our GitHub repository (https://github.com/jiangting368/Ppara_enhancer_analysis).

### Download and analysis of public ChIP-seq, ATAC-seq, and GRO-seq

The ATAC-seq, ChIP-seq, and GRO-seq datasets used in this study were obtained from the EBI ENA database (https://www.ebi.ac.uk/). For GRO-seq analysis, a modified analysis was implemented according to the previous method [58,59]. Briefly, low-quality bases, tailing polyA, and adapter sequences were trimmed using Cutadapt (v3.3). Trimmed reads were aligned to the mm10 mouse genome using Bowtie (v1.0.0) with parameters ‘-n 2 -l 32’. We employed HOMER (v4.11) to generate BedGraph files, then converted into BigWig files using bedGraphToBigWig (v4) and visualized using Integrative Genomics Viewer (IGV, v2.10.0). All software commands and workflow configuration files used for GRO-seq data analysis have been deposited in our GitHub repository (https://github.com/jiangting368/Ppara_enhancer_analysis). For ATAC-seq and ChIP-seq data, peak calling was conducted using MACS2 (v2.2.7.1) and visualized using IGV (v2.10.0). The download date utilized in this study is list in S6 Table.

### Identification of active enhancer of *Pparα* gene

Genomic regions showing significant chromatin interaction with the **Ppar*α* promoter and at least 2000 bp upstream TSS of **Ppar*α* were considered potential enhancers. Chromatin interactions of the **Ppar*α* promoter were first examined by 4C-seq, which provided potential regions with physical contacts [35]. To further prioritize putative enhancers, these potential regions were intersected with published epigenomic and transcriptomic datasets. Specifically, potential regions were required to show enrichment of H3K27ac and H3K4me1 [16], chromatin accessibility from ATAC-seq [60], and exhibit transcriptional activity reflected by RNA polymerase II recruitment and eRNA transcription detected by GRO-seq [61,62]. Applying these criteria, five putative enhancers were selected for subsequent analyses.

The putative active enhancer fragments were inserted into pGL3-Promoter reporter vector (Promega). On day 6 of differentiation, brown adipocytes were transfected with pGL3-Promoter-enhancer luciferase vector using Lipofectamine 3000 (Invitrogen). After 48 h, luciferase activity was measured using Dual-Luciferase Assay Kit (Vazyme, Nanjing, China). The primer sequences of enhancer are provided S9 Table.

### Design of sgRNAs and plasmid construction

All sgRNAs were designed using CHOPCHOP [63] (http://chopchop.cbu.uib.no/) and CRISPOR [64] (http://crispor.tefor.net/). To assess genome-wide specificity, potential sgRNAs were further evaluated using Cas-OFFinder [65] (http://www.rgenome.net/cas-offinder/) and CRISPR Finder [66] (https://wge.stemcell.sanger.ac.uk/). To ensure efficient transcription for U6 promoter, each sgRNA sequence should start with a ‘G’. Annealed oligonucleotides were then ligated into the linearized pLV hU6-sgRNA-PGK-puromycin vector (Addgene) with BsmBl-v2 site using a DNA Ligation Kit (Takara). The sequences of Pparα-En4 sgRNAs primers are list in S5 Table.

### Mouse brown preadipocytes cultures and differentiation

Primary brown preadipocytes were isolated from iBAT of C57BL/6J male mice aged 5 weeks with using collagenase Ⅰ, as previously established [67]. Briefly, iBAT were aseptically dissected, and mechanically dissociated into 1–2 mm^3^ fragments. Next, tissue fragments were enzymatic digestion in 0.1% collagenase Ⅰ solution (w/v) at 37°C for 50 min. The cell suspension was further filtered through 40 μm cell strainer and plated into 12-well plate, maintained in 5% CO_2_ at 37°C. Upon reaching confluence, primary brown preadipocytes were cultured in induction DMEM/F-12 medium containing 10% FBS, 850 nM insulin (MCE), 0.5 mM isobutylmethylxanthine (IBMX), 5 μM dexamethasone,1nM T3 and 1 μM rosiglitazone (all from Sigma-Aldrich). Two days later, the medium was replaced by differentiation medium containing 850 nM insulin, 1 nM T3 and 1 μM rosiglitazone. The brown adipocytes were harvested on the eighth day.

### Immunofluorescence and BODIPY staining

Differentiated adipocytes were fixed using 4% paraformaldehyde for 15 min at RT, and then permeabilization with 0.2% Triton X-100. Subsequently, cells were blocked for 30 min at 37°C with 10% normal goat serum. For immunostaining, the primary and secondary antibodies are rabbit anti-PPARα (Abclone, Wuhan, China) and Cy3 goat anti-rabbit IgG (Abclone). Nuclear counterstaining was performed using DAPI (Beyotime, Shanghai, China) for 5 min. For BODIPY staining, adipocytes were staining with 1µM BODIPY 493/503 (Thermo Fisher) for 20 min. Fluorescence images were acquired using an Olympus IX73 inverted microscope (Olympus) and image analysis was conducted using ImageJ.

### RNA extraction and qRT-PCR

Total RNA was extracted using Trizol reagent (Invitrogen) and subsequently reverse transcribed into complementary DNA with the HiScript III RT SuperMix (Vazyme). Quantitative real-time PCR (qRT-PCR) was carried out on a CFX Connect Real-Time System (Bio-Rad) employing the ChamQ Universal SYBR qPCR Master Mix (Vazyme). Relative expression levels of target genes were calculated by the 2^-ΔΔCt^ method [68], with *Rplp0* as the reference gene [69]. The primer sequences are provided in S8 Table.

### Mitochondrial DNA content

Total DNA was extracted using Genomic DNA kit (TIANgen, Beijing, China). Mitochondrial DNA (mtDNA) content was assessed as the ratio of the copy number from the mtDNA-encoded gene (16S) to the nuclear-encoded gene (*Rplp0*). The primer sequences are provided in S8 Table.

### Western Blotting

Protein was extracted using BBproExtra Total protein extraction kit (Beibokit, Shanghai, China), and its concentration was measured using the BCA protein assay (Beibokit). Protein samples were separated on 10% SDS-PAGE gels and transferred to PVDF membranes. After blocking, membranes were incubated overnight at 4°C with the following primary antibodies: rabbit anti-Cas9 rabbit (1:1000, Cell Signaling Technology), rabbit anti-UCP1 (1:1000, Cell Signaling Technology), mouse anti-PPARα (1:2000, Proteintech, Wuhan, China), total OXPHOS cocktail (1:1000, Abcam), and rabbit anti-Vinculin (1:2000, Cell Signaling Technology). Membranes were then incubated with HRP conjugated anti-mouse or anti-rabbit IgG (HUABIO, Wuhan, China). Finally, the PVDF membranes were visualized with an automatic chemiluminescence apparatus (Bio-Rad) and quantified using ImageJ.

### DNA pulldown assay

Pparα-En4 sequences were obtained by PCR using 5’-biotin-Pparα-En4 primers. The forward sequence: AGGAGGTAAAGCCACAAGCC, the reverse sequence: CACCATCCCAGAGCTAACCC. Interscapular BAT from male C57BL/6J aged 8 weeks (10 mice per replicate) were divided into control and experiment groups, each with two replicates. The control group included magnetic beads without non-labeled DNA. DNA pulldown assay was conducted utilizing a DNA pulldown kit (BersinBio, Guangzhou, China). Sliver staining was employed to visualize the pulldown proteins, which were analyzed by Q-exacitve Plus mass spectrometer (Thermo Scientific). The function and annotation of the proteins were obtained from Uniport (https://www.uniprot.org/). Mass spectrometry data were analyzed using MaxQuant (version 2.1.2.0) with the integrated Andromeda search engine against the UniProt mouse database (March 2023). The false discovery rate (FDR) was controlled at less than 1% for both proteins and peptide-spectrum matches.

### ChIP assay

Chromatin immunoprecipitation (ChIP) assays were performed using commercial kits (Beyotime for cultured cells; GENE CREATE for tissues). For in vitro experiments, brown adipocytes were transfected with CREB overexpression vector on day 6 of differentiation. Two days later, adipocytes were crosslinked by incubation with 1% formaldehyde for 10 min at RT to preserve protein-DNA interactions, followed by quenching with glycine. For in vivo assays, interscapular BAT from 8-week-old male C57BL/6J mice was processed similarly. Chromatin was then sheared by sonication using a Q800R3 Sonicator (QSonica, Newtown, USA) to generate DNA fragments ranging from 200 to 1000 bp. For immunoprecipitations, 2 μg of antibodies against phospho-CREB (Ser133, Cell Signaling Technology), CBP (Cell Signaling Technology) or IgG control were incubated with the protein-DNA complex overnight at 4°C. qRT-PCR was used to detect Pparα-En4 enrichment. Primer sequences are provided in S8 Table.

### Construction of vectors and luciferase reporter assay

Protein coding regions of CREB, ATF1, CREM and CBP were inserted into linearized pEGFP-N1 vector at the *Kpn*Ⅰ restriction site. PGL3-Promoter-Pparα-En4 (50 ng) and/or each overexpression vectors (50 ng) were co-transfected with into H293T cells using Lipofectamine 3000 (Invitrogen). After 48 h, Luciferase activity was performed using the Dual-Luciferase Reporter Assay System. Cells were fixed using 4% paraformaldehyde for 15 min at RT, and then permeabilization with 0.2% Triton X-100. Nuclear counterstaining was performed using DAPI (Beyotime) for 5 min. Primer sequences are provided in S9 Table.

### Lentivirus production

HEK293FT cells were used to produced lentivirus. Cells were co-transfected with 48 μg of the pLV-hU6-sgRNA-hUbC-dCas9-KRAB-T2a-GFP, 12 μg of pVSV-G, and 35 μg of psPAX2 (all from Addgene) employing a Calcium Phosphate Transfection Kit (Beyotime). Medium containing lentivirus were collected at 24, 48, and 72 h post-transfection subsequently concentrated using Amicon Ultra-15 100 kDa centrifugal filter units (Millipore). Viral titers were detected using a colloidal gold kit (Biodragon, Beijing, China).

### In vivo lentiviruses injection into iBAT

Five-week-old male C57BL/6J mice were housed under either thermoneutral (30°C) or cold (4°C) conditions with unrestricted access to standard rodent chow and drinking water, and a standardized 12 h light/dark cycle. Within each temperature condition, mice were randomly divided into two experimental groups (n = 5 per group): one receiving dCas9-KRAB-GFP lentivirus (control group) and the other receiving dCas9-KRAB-Pparα-En4 lentivirus (treatment group). One week later, mice were anesthetized by 1% sodium pentobarbital. Following previously described methods [70], each animal was received 200 μL of lentivirus particles (6 × 10^7^ lentiviral transducing particles (TU)/mL), injected into iBAT depot at four injection sites per side. Mice were received four lentiviral injections, once every three days. For mice exposed to cold, mice were given a 12 h recovery period at 25°C post-injection before being transferred to 4°C. Following the final injection, all mice were maintained under a 16-day acclimatization period under their respective thermal conditions (30°C or 4°C) prior to terminal tissue collection.

### Histological staining

Adipose tissues were fixed in 4% paraformaldehyde before dehydration and paraffin embedding. For histological examination, sections were prepared and stained using conventional hematoxylin and eosin (H&E) protocols to visualize tissue structure.

### Infrared and fluorescence imaging

Prior to imaging, interscapular fur was removed by shaving. Thermal activity of iBAT was assessed using an infrared thermography system (Fotric 348A, Shanghai, China). Thermal images were subsequently processed with Analyz IR. Lentiviral transduction efficiency and spatial distribution were validated via fluorescence imaging using the AniView Kirin Small Animal in vivo 3D Imaging System (Biolight, Guangzhou, China)

### Transmission electron microscopy

Mice iBAT samples were fixed for 2 h in electron microscope fixative (Servicebio) After dehydration with ethanol and acetone dehydration, the tissues were embedded and sliced into ultrathin sections approximately 60 nm. These sections were then contrasted with imaged acetate and lead citrate before being examined using a JEM 1400 Transmission Electron Microscope (JEOL Ltd).

### Statistical analysis

Quantitation data are expressed as mean ± standard error of mean (SEM). For comparisons between two independent groups, unpaired two-tailed Student’s t-tests were employed. In cases involving multiple group comparisons, two-way analysis of variance (ANOVA) was conducted, followed by Tukey’s post hoc test to assess pairwise differences. The *p*-value < 0.05 was considered indicative of statistical significance.

## Supporting information

S1 Fig Construction and quality control of the 4C-seq library. (A) Viewpoint selection and primer design at **Ppar*α* promoter region between 2000 bp upstream to 500 bp downstream of the transcription start site for the 4C-seq experiment. (B) Bar plots showing the percentage of mapped reads in *cis*-chromosome and *trans*-chromosome for each 4C dataset.
(TIF)

S2 Fig Expression pattern of *Ppar*α in brown adipocyte differentiation. (A) Expression pattern **Ppar*α* was confirmed by qRT-PCR in brown adipocytes during differentiation. (B) Expression of **Ppar*α* was confirmed by qRT-PCR in brown adipocytes treated with ISO (10 μM) for 4 h (n = 6). (B) Amplification curve of *dCas9* and *Rplp0* (left) and melting curve of *dCas9* (left) and *Rplp0* (right) in brown adipocytes. Data are shown as mean ± SEM. (D) Western blot (n = 3) analysis of Cas9 expression. (E) qRT-PCR analysis of genes related to brown adipocyte function (*Abhd5*, *Cidea*, *Dio2*) (n = 6). Data are shown as mean ± SEM.
(TIF)

S3 Fig Transfection efficiency of overexpression transcription factors. (A) GFP fluorescence imaging of transfection with eGFP_N1 constructs in 293T cells. (B) Expression of *Crebbp*, *Atf1*, *Crem* and *Creb* was confirmed by qRT-PCR in transfection with CBP, ATF1, CREM and CREB over expression constructs in 293T cells (n = 6). Data are shown as mean ± SEM.
(TIF)

S4 Fig Repression of Pparα-En4 does not impair thermogenic capacity or mitochondrial integrity in iBAT at TN. (A) Representative images of iBAT in mice two weeks after injection of dCas9-KRAB-GFP lentivirus. (B) GFP expression (no antibody staining, green) and nuclei (blue) are shown in iBAT. Scale bar = 100 μm. (C, D) qRT-PCR (n = 5) and western blot (n = 3) analysis of PPARα expression, band intensity was analyzed using ImageJ. (E) Body weight of control and iBAT-En4 mice at CE (left) or TN (right) (n = 5) (F) Western blot analysis of UCP1 (n = 3), band intensity was analyzed using ImageJ software. (G) qRT-PCR analysis of genes related lipolysis-related genes (*Lipe*, *Mgll* and *Pnpla*). (H) TEM images of iBAT mitochondrial at TN. Scale bar = 2 μm. (I) mtDNA quantification by qRT-PCR in iBAT, comparing control and iBAT-En4 mice at TN (n = 5). (J) Western blot analysis of OXPHOS complex subunits in iBAT at TN (n = 3). Band intensity was analyzed using ImageJ. Data are shown as mean ± SEM.
(TIF)

S5 Fig Uncropped western blot images. (TIF)

S1 Table The quality metrics of 4C-seq data. (XLSX)

S2 Table Genome-wide chromatin interaction sites of 4C data identified by r3Cseq. (XLSX)

S3 Table Chromatin interaction sites between replicates identified by r3Cseq. (XLSX)

S4 Table The PCR primers for 4C-seq library construction. (XLSX)

S5 Table CRISPRi sgRNA for targeting active enhancers of *Pparα.* (XLSX)

S6 Table The detailed information of ChIP-seq, ATAC-seq, DHS-seq data, and GRO-seq. (XLSX)

S7 Table Mass spectrometry of Pparα-En4 pulldown proteins. (XLSX)

S8 Table The primers for gene expression, mtDNA copy number, and ChIP-qPCR. (XLSX)

S9 Table The PCR primers of constructed vectors. (XLSX)

S10 Table The values used to build graphs in the paper. (XLSX)
