# Supplementary figures and images for: A distal enhancer of Pparα regulates thermogenesis and mitochondrial function in brown fat
Source: PLoS Genet. 2025 Oct 23;21(10):e1011915. doi: 10.1371/journal.pgen.1011915 (PMC12574865; doi:10.1371/journal.pgen.1011915)

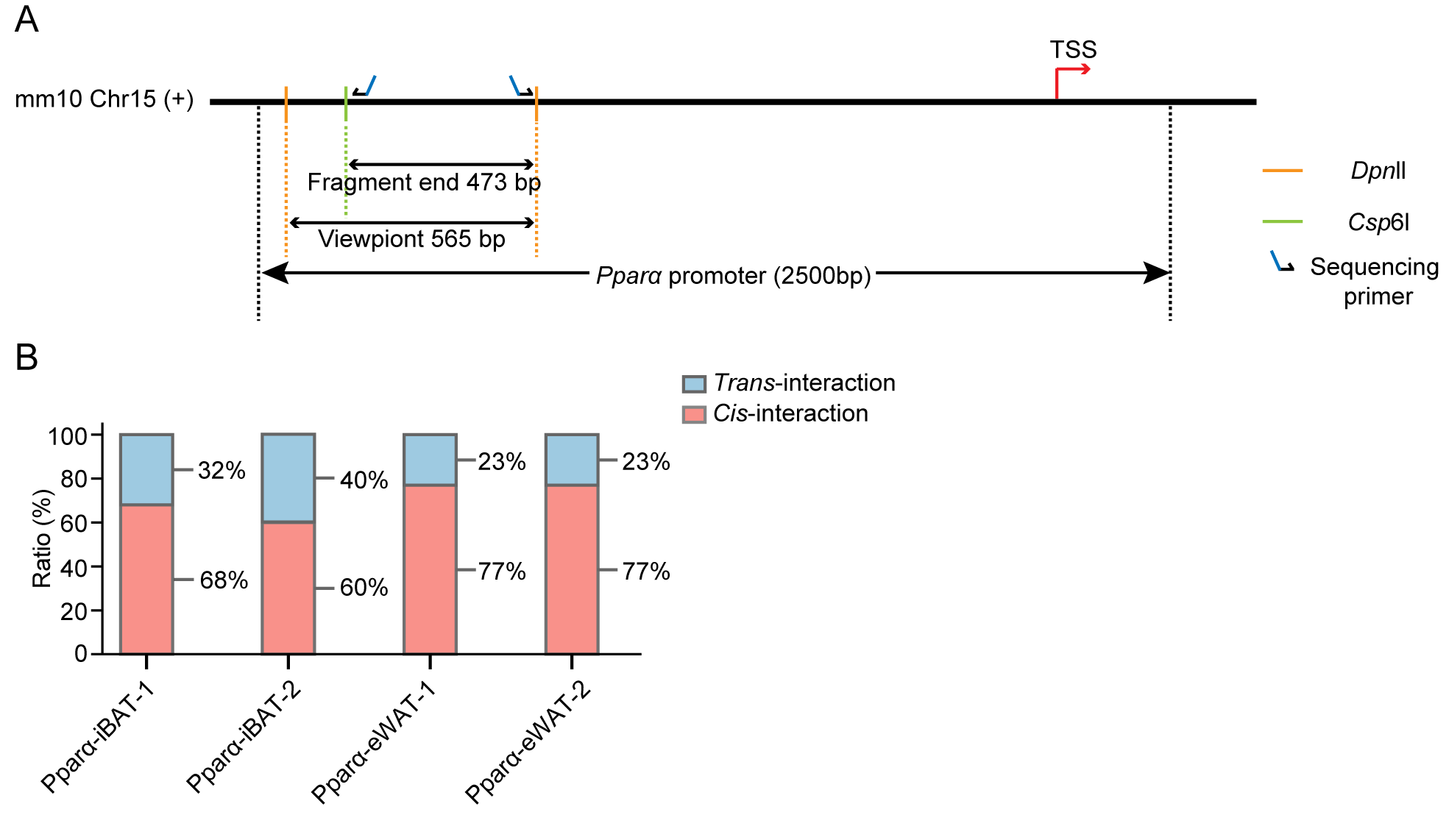

Supplement: S1 Fig — (A) Viewpoint selection and primer design at Pparα promoter region between 2000 bp upstream to 500 bp downstream of the transcription start site for the 4C-seq experiment. (B) Bar plots showing the percentage of mapped reads in cis-chromosome and trans-chromosome for each 4C dataset. (TIF) [file pgen.1011915.s001.tif]

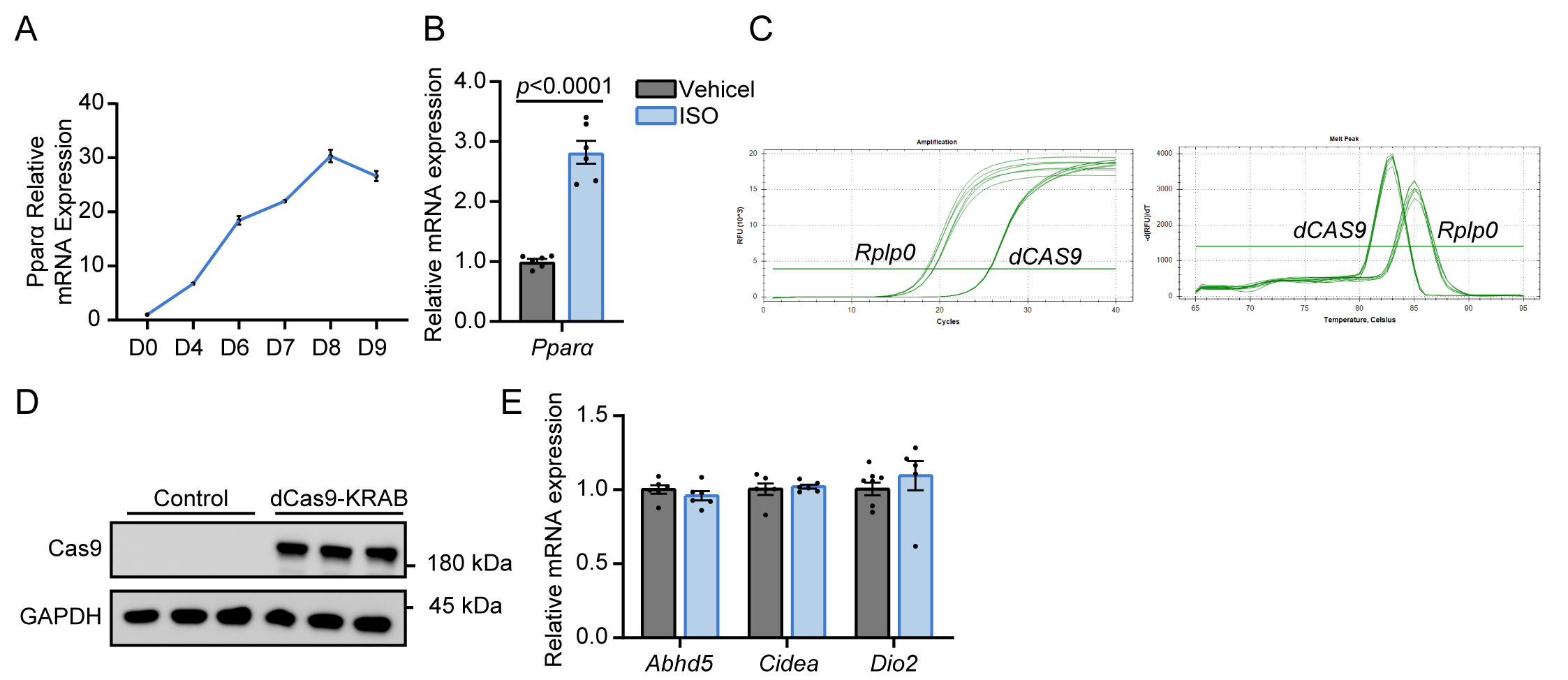

Supplement: S2 Fig — (A) Expression pattern Pparα was confirmed by qRT-PCR in brown adipocytes during differentiation. (B) Expression of Pparα was confirmed by qRT-PCR in brown adipocytes treated with ISO (10 μM) for 4 h (n = 6). (B) Amplification curve of dCas9 and Rplp0 (left) and melting curve of dCas9 (left) and Rplp0 (right) in brown adipocytes. Data are shown as mean ± SEM. (D) Western blot (n = 3) analysis of Cas9 expression. (E) qRT-PCR analysis of genes related to brown adipocyte function (Abhd5, Cidea, Dio2) (n = 6). Data are shown as mean ± SEM. (TIF) [file pgen.1011915.s002.tif]

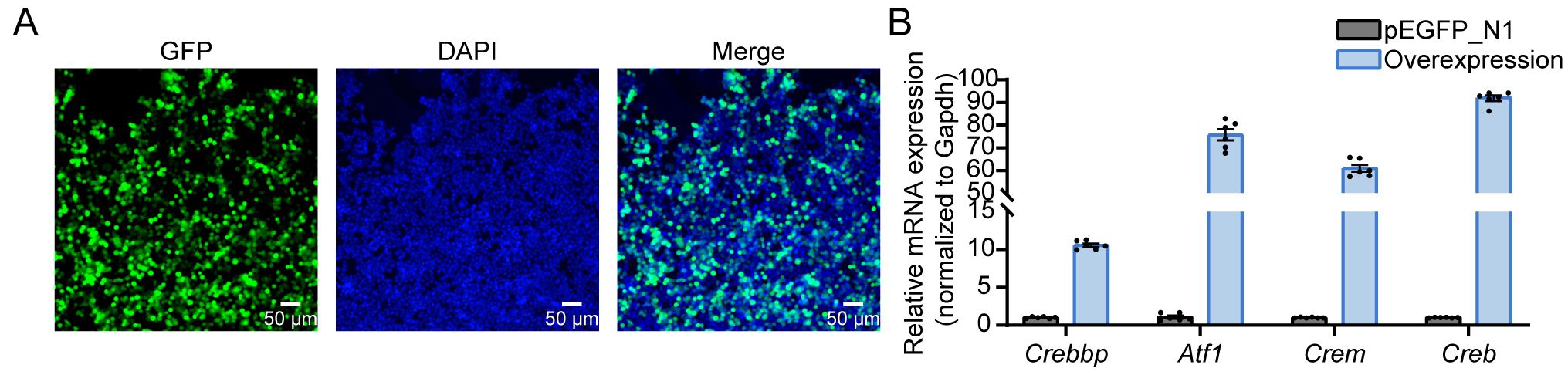

Supplement: S3 Fig — (A) GFP fluorescence imaging of transfection with eGFP_N1 constructs in 293T cells. (B) Expression of Crebbp, Atf1, Crem and Creb was confirmed by qRT-PCR in transfection with CBP, ATF1, CREM and CREB over expression constructs in 293T cells (n = 6). Data are shown as mean ± SEM. (TIF) [file pgen.1011915.s003.tif]

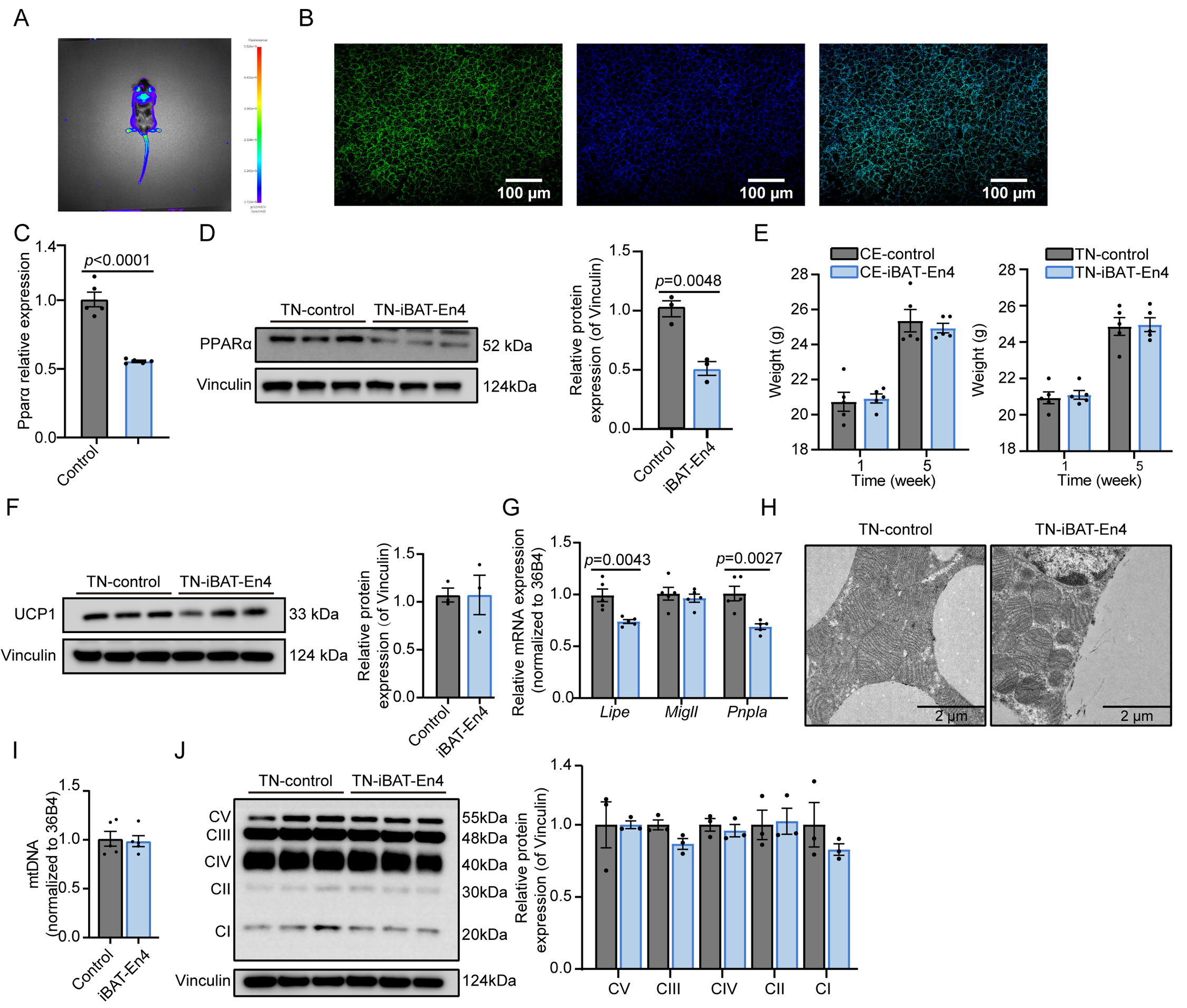

Supplement: S4 Fig — (A) Representative images of iBAT in mice two weeks after injection of dCas9-KRAB-GFP lentivirus. (B) GFP expression (no antibody staining, green) and nuclei (blue) are shown in iBAT. Scale bar = 100 μm. (C, D) qRT-PCR (n = 5) and western blot (n = 3) analysis of PPARα expression, band intensity was analyzed using ImageJ. (E) Body weight of control and iBAT-En4 mice at CE (left) or TN (right) (n = 5) (F) Western blot analysis of UCP1 (n = 3), band intensity was analyzed using ImageJ software. (G) qRT-PCR analysis of genes related lipolysis-related genes (Lipe, Mgll and Pnpla). (H) TEM images of iBAT mitochondrial at TN. Scale bar = 2 μm. (I) mtDNA quantification by qRT-PCR in iBAT, comparing control and iBAT-En4 mice at TN (n = 5). (J) Western blot analysis of OXPHOS complex subunits in iBAT at TN (n = 3). Band intensity was analyzed using ImageJ. Data are shown as mean ± SEM. (TIF) [file pgen.1011915.s004.tif]

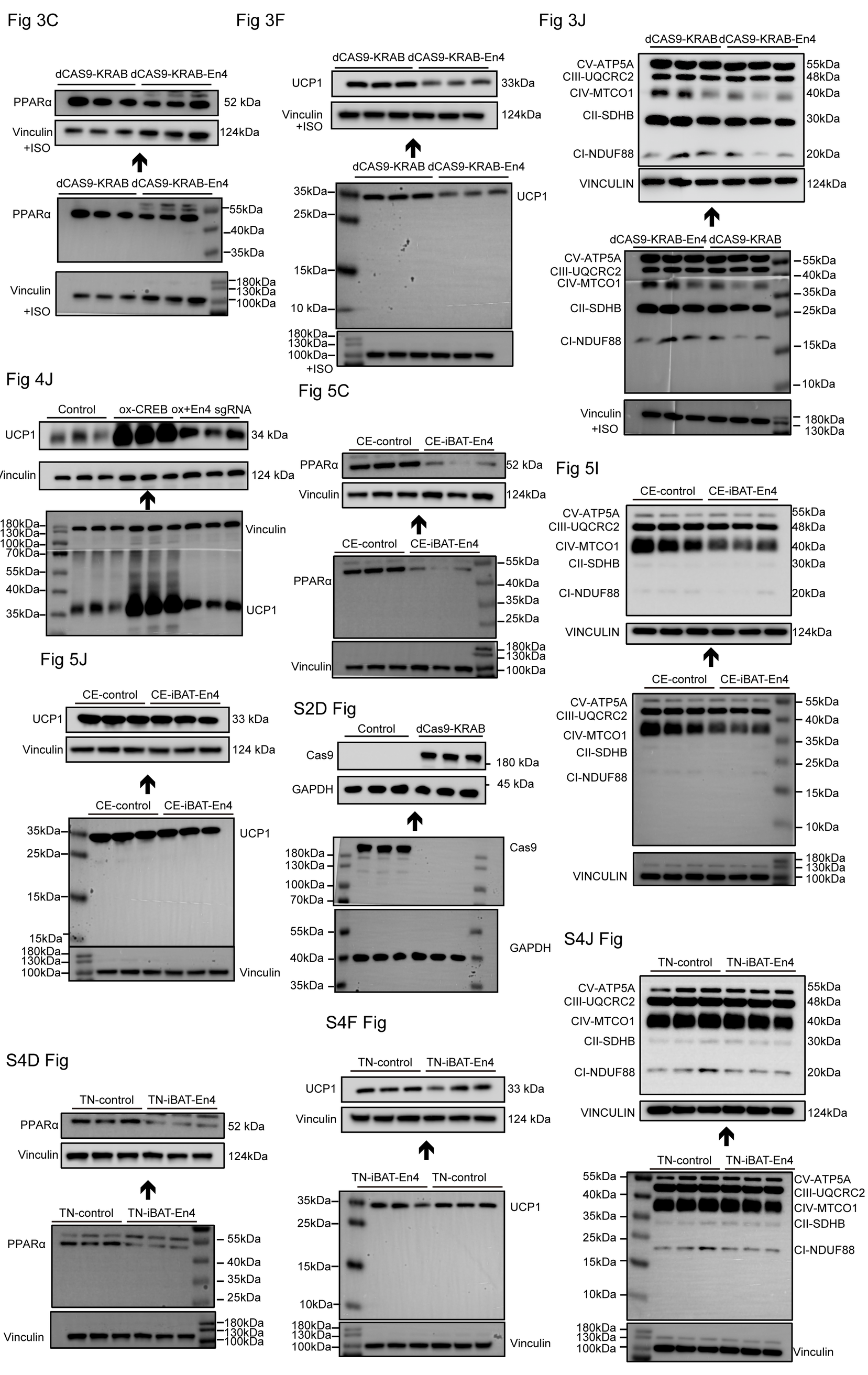

Supplement: S5 Fig — (TIF) [file pgen.1011915.s005.tif]
